# An Improvised Machine Learning Model Based on Mutual Information Feature Selection Approach for Microbes Classification

**DOI:** 10.3390/e23020257

**Published:** 2021-02-23

**Authors:** Anaahat Dhindsa, Sanjay Bhatia, Sunil Agrawal, Balwinder Singh Sohi

**Affiliations:** 1 Department of Electronics and Communication Engineering, Chandigarh University, Gharuan, Punjab 140413, India; bssohi@yahoo.com; 2 University Institute of Engineering and Technology, Panjab University, Chandigarh 160014, India; s.agrawal@hotmail.com; 3 Post Graduate Department of Zoology, University of Jammu, Kashmir 180006, India; nitujmu2002@yahoo.co.in

**Keywords:** mutual information, classification, k-fold cross validation, machine learning modeling, image segmentation, microorganisms

## Abstract

The accurate classification of microbes is critical in today’s context for monitoring the ecological balance of a habitat. Hence, in this research work, a novel method to automate the process of identifying microorganisms has been implemented. To extract the bodies of microorganisms accurately, a generalized segmentation mechanism which consists of a combination of convolution filter (Kirsch) and a variance-based pixel clustering algorithm (Otsu) is proposed. With exhaustive corroboration, a set of twenty-five features were identified to map the characteristics and morphology for all kinds of microbes. Multiple techniques for feature selection were tested and it was found that mutual information (MI)-based models gave the best performance. Exhaustive hyperparameter tuning of multilayer layer perceptron (MLP), k-nearest neighbors (KNN), quadratic discriminant analysis (QDA), logistic regression (LR), and support vector machine (SVM) was done. It was found that SVM radial required further improvisation to attain a maximum possible level of accuracy. Comparative analysis between SVM and improvised SVM (ISVM) through a 10-fold cross validation method ultimately showed that ISVM resulted in a 2% higher performance in terms of accuracy (98.2%), precision (98.2%), recall (98.1%), and F1 score (98.1%).

## 1. Introduction

Biodiversity informatics [1,2] is an emerging field that has found a high degree of attention in today’s context. Due to climate change, there is an urgent need to review the management of the ecological resources of an area. The domain of biodiversity informatics [3] is an application of computer-based operations, functions, algorithms, and techniques that help to organize data, conduct environment sampling for computing biodiversity indices. This is done so that the impact on living organisms can be assessed due to changes in the population of species and climate or both. Hence, through this study, we intend to automate the procedures where these assessments can be done with the help of computer aided algorithms. Today, the conservation of native species in agriculture, ocean, fisheries, forestry, etc. is considered one of the main goals of bioinformatics. Activities such as species identification and mapping [4] of the biodiversity of an area are an essential part of biodiversity activities. There are multiple organizations that are maintaining records on the taxonomy, genes, and population of the species and have developed tools to visualize the biodiversity data [5]. With the advancements in the Internet of Things (IOT) [6] and microscopic imaging, the scope of bioinformatics has increased many folds. Further, with the help of the microscopic level of photography, many researchers, organizations, and governments are mapping the biodiversity of microorganisms which was not possible earlier [7]. Due to this, studies regarding invasive species at the microscopic level are possible now [8]. The microscopic imaging has also given birth to computational algorithms that count, group, and identify the microorganisms automatically.

The identification of the microorganism is a tedious task [9]. There is always a need for an expert and a person who understands the nuances of taxonomy and other characteristics of microbes such as odor [10]. With the emergence of machine learning models and statistics, now the process of identification can be automated with a high degree of accuracy [11], and assessment regarding biodiversity [12] can be automated. 

At the microscopic level, different researchers have done specialist work in biodiversity. The main focus is to work on the biodiversity of algae [13,14] as they are indicators of water quality and health of the water bodies. Few are also working on biodiversity studies on microscopic fungi [15,16]. Research on biodiversity of bacteria [17] in water bodies has a lot of citations in high impact journals. Most of the work has been done in the context of isolation, semi-automatic/automatic identification, and classification of the algorithms. All of the microscopic organisms are isolated using well established methods and laboratory protocols. With the passage of time, identification and classification of the microorganism tasks are shifting towards automation [18]. 

Recent studies [9,19,20] are focusing on automating the process of identification with the help of image processing and machine learning models. The purpose of image processing is to effectively isolate the microorganism body from the images. Thresholding [21] methods are being used by researchers either as the main function to segment or as an auxiliary function for microbe segmentation. The survey [22] shows that image thresholding and level set-based algorithms to segment the bacteria are being used. Biological studies have widely used image thresholding and level set-based algorithms in various other applications such as lung segmentation, tissue extraction, and protein synthesis, etc. [23,24,25].

There are three most frequently used methods for segmentation of the microorganisms. The first one is the edge-based method, the second one is based on threshold as mentioned earlier, and the third one is the region-based method such as region growing. The use of machine learning and an optimization algorithm to segment the image is also popular. Many researchers are doing microbe segmentation using convolution architectures networks such as U-net, FCN (fully convolutional networks), and VGG (visual geometric group), etc. Traditional unsupervised algorithms such as K- means, c-means, fuzzy c-clustering have also been used for microbes segmentation. It should however be noted that most of these research works focus on a limited/specific number of species. The post processing i.e., after the microbe segmentation, the segmented images are subjected to the feature extraction [26] and selection [27] process. Recent developments [22,28,29] are based on extracting morphological features for building the identification system. Few studies [30] are using texture features for understanding the body shapes and pattern of the texture of the microbes. In summary, it can be said the most common features that researchers are using include shape, geometric, spectrum [30], and color-based [31] features.

Studies limited to particular species for automatic identification can be found in most of the current journals [32,33,34]. Artificial neural network (ANN) is being used for automating the tasks of identification of bacteria [35]. Many studies are applying KNN for identification of the microbial species. The SVM algorithm is a widely used method of classification. There is evidence that probability classifiers are also in use for identifications of microbial species and the outcome in terms of average accuracy is above 90%. Studies specific to the particular zone (river basin [36], littoral, benthic [7,37,38]) of the water bodies have also been found in the current literature. 

This research initiative was taken to construct a fully automated system that can identify and classify microorganisms. As per the research scope, the microbes belong to the surface water zone and they are analyzed with the help of microscopy imaging. The fully automated system includes two main components; imaging and classification. The focus is to create a generic pipeline of algorithms for automating the identification process. Last but not least, the work is not limited to the analysis of previous works but also includes improvements and improvisations for achieving the said goal. The level of performance obtained is comparable to deep learning algorithms used by other authors for a similar purpose [35,39,40].

In terms of technical contribution, this original research work demonstrates the construction of a novel microbe segmentation algorithm and an improvised automation process for accurately classifying ten microbes. As per our best knowledge, such elucidation and implication for classifying multiple species has not been done till date, especially in the context of biodiversity studies of Chandigarh water bodies, India.

The paper is organized as follows: After the introduction, Section 2 gives the methodology on how the goals of the study are achieved. Sample collection and description of the slide preparation are discussed here. In addition, the characteristics of the image dataset are mentioned. The next steps; image segmentation and machine modeling are explained in detail under this section. Section 3 and Section 4 cover results and discussions respectively.

## 2. Materials and Methods

**Figure 1:**
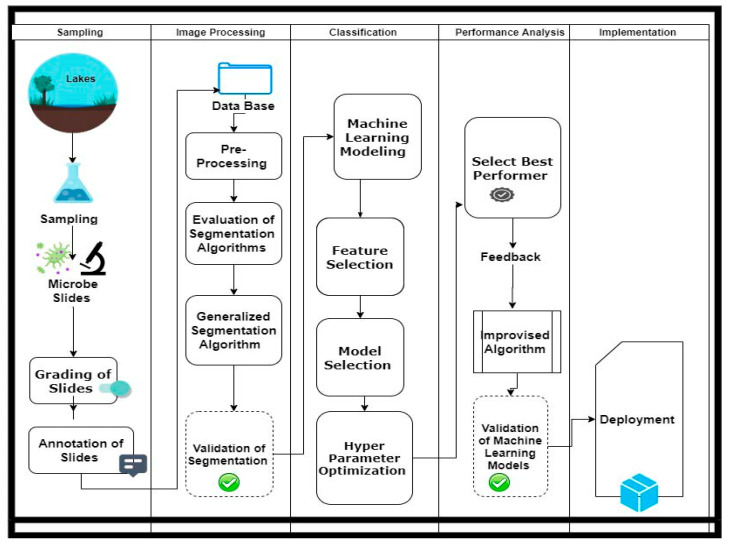
Research flow.

The methodology is divided into three steps. The first step is to collect primary data. The second step is to acquire accurate boundaries of microorganisms by using multiple image processing operations. The third step is to select the best features for classification and further select the best machine learning models for automation. Figure 1 gives a stepwise illustration on how the research work is executed.

### 2.1. Sampling

The water samples were collected from the surface areas of two water bodies (Sukhna and Dhanas Lakes), Chandigarh, India [41]. Sukhna Lake [42] is a water catchment area (25 square kilometers) of Shivalik foothills, hence is fed by rain along with seasonal streams. Dhanas Lake [43] is a man-made water body to capture rainwater from adjacent areas. Fifteen sampling points were selected for each water body. For capturing microscopic images of the microorganism, slides were prepared using different concentrations of ethanol with the sample water. This was done until a whole mounted image could be taken.

Approximately three thousand microbe slides for both lakes (magnification 4× and 10×) were made and microscopic images were captured from these slides. The microscope used in this work had achromatic 4× to 100× objectives, coaxial coarse, and fine adjustment capacities, and a focusing range up to 30 mm with 0.002 mm focusing interval. The work of quality, grading, and labeling each slide as per the species was done with the help of a domain expert in order to maintain the semantic correctness, syntactic correctness, consistency, completeness, and uniqueness of the data. The final dataset of 600 images (whole mounted) was further subjected to processing. Out of these 600 images, a number of microorganisms were identified wherein each microorganism represented a data instance. 

Table 1 gives a list of microorganism classes that were used to label data sets for training machine learning models. It can be observed that Volvox has 7002 instances and so on. Furthermore, it can be calculated that the total number of instances is 32,779.

### 2.2. Image Processing

This section elaborates the various steps taken to prepare the machine learning data from the images. A file-based data repository was built for further performing the image processing operations. Each microorganism has a separate folder and metafile for maintaining the record. It is always desirable that highly accurate and relevant data should be fed to an automated system for its stability. Hence, preprocessing is a precursor for constructing such systems. The extraction of microbe bodies consists of the following steps.

#### 2.2.1. Image Preprocessing

The quality of the image depends on the skills of the person making slides and the one who is capturing images using a microscope [44]. Hence, the following steps were taken to enhance the quality and variability of the dataset.

- Aspect Ratio: All images were standardized into aspect ratio (1:1.2) so that computation of features such as center of mass of the region of interest does not deviate far away from the normal trend of values.
- Area-based Object Removal: Some images had unwanted objects such as debris etc. Removal of objects fewer than a configurable size value was done with the help of a custom filter.
- Irregular Object Removal: Highly deformed or highly irregular shapes of the objects were identified and filtered so that only useful information is left within the semantics of the image.
- Noise Removal: The median filter was applied to remove any other noise that may be left after applying the steps listed above.
- Contrast Enhancement: Adaptive contrast enhancer [45] was used to increase the overall difference of intensities so that the segmentation algorithm finds it easy to process.

#### 2.2.2. Segmentation

For the identification and extraction of microorganism’s boundaries, seven convolution gradient filters were evaluated as shown in Table 2. The experimentation process was validated with the help of Intersection over Union (IoU) metric [46]. IoU is a metric that calculates overlap between the benchmarked images and the image created by convolution gradient filters, based on the threshold of 0.5. Random sampling has been used for each filter so that the bias is minimized while calculating the average IoU. It can be observed that for a sample size of 25, the Kirsch filter performs the best followed by Prewitt and mean filters. As the sample size increases, Kirsch filter continues to give the best performance. Consequently, the averages of filters show that Kirsch filter’s accuracy (0.90) is the highest based on IoU metric.

The next step is to cluster all those pixels that can form the boundary of microorganism and improve the quality of segmentation output. This will help to improve the accuracy of classification algorithms. A comparative study between the ISODATA clustering [51] and Otsu method [52] revealed that the Otsu method is more accurate in helping to group the pixels of the microorganisms to form the shape. This was done using subjective evaluation with the help of an expert by using a random sampling method. Multiple sets of samples were chosen randomly. Further, each set had randomly chosen images. The accuracy (Accepted and Correct ‘AC’ Segmented/Sample Size, ‘S’) based on the current sample size and with respect to the full dataset was computed as shown in Table 3. 

#### 2.2.3. Generalized Segmentation Algorithm (GSA)

This section gives the logical statements of all the steps used in conducting the image processing to achieve the aforementioned objectives. GSA is summarized in Algorithm 1.

**Algorithm 1:** Generalized Segmentation Algorithm **Input:** Set of Microscopy Images, ‘MI’, Global Intensity Threshold (git), Tile Size = Ts //window size. Kirsch_Filter_Mask = {Gx, Gy}, {Gx, Gy}, {Gx, Gy}, {Gx, Gy}, {Gx, Gy}, {Gx, Gy}, {Gx, Gy}, {Gx, Gy} **Output:** Microorganism Shape Matrix: ‘BO’ **1** Initialize Variables: Path of Microscopy Images ‘MD’ folder, filename = ‘f’, Counter ‘C’= 0 **2** Compute global standard deviation: for each Microscopy Image file: ‘MI’ in ‘MD’     Irbg = Read Image Matrix (I)     Irg = Normalize, Resize Image Matrix (Irgb)     Gray = Gray_Scale(Irg)     Gray_Std = Compute_Global_Std(Gray) **3** Segment micoorganism: If Gray_Std (< Global_Std) {       G = Apply_Adaptive_Enchancer (Gray) CF = Run_2D_ Kirsch_Filter(Kirsh_Filter_Mask,G)  //Convolution Filter BO = Global_Thresholding(git);   } else { CF = Run_2D_ Kirsch_Filter(G)  //Image Derivative Filter BO = OTSU_Global_Thresholding();     C = C + 1 }End for 

The output of GSA is shown in Table 4. After the completion of image segmentation, the images are then subjected to the feature extraction process required for building an automated system for identification and classification of microbes. The extracted features include Solidity, Eccentricity, EquivDiameter, Extrema [53], Filled Area, Extent, Orientation, Euler number [54], Bounding box [55], Convex hull, Major and Minor axis [56], Perimeter, Centroid, Area [57], convex area [58], and radii [58]. In total, twenty-five attributes are constructed, where the last attribute represents the class. These attributes make a feature row. Each feature row represents the properties of a blob. Blob is basically the unit based on which the shape of the microorganism can be understood. These attributes were used for training machine learning models. The dataset and its related information on all the attributes is available in the repository [59]. The next section gives detailed information on the process of automating the tedious task of manual identification. 

### 2.3. Classification

Machine model selection is a process of evaluation of machine learning models and their parameters specific to the goals of automation. The process includes evaluation of different sets of data (training, testing, and validation) and fine tuning with hyperparameters. In the context of this research work, we have done extensive hyperparameter tuning using random grid search methods on each machine learning model. For a better understanding of the evaluation parameters, Table 5 can be referred.

Five algorithms: multi-layer perceptron (MLP) [30,62], K-nearest neighbors (KNN) [63,64], quadratic discriminant analysis (QDA) [33], logistic regression (LR) [64,65], and support vector machine (SVM) [65,66] are evaluated in terms of accuracy, precision, and recall performance metrics as the main criteria for finalizing the hyper-parameters and model selection. The selection of classifiers is based on the hypothesis that one of these algorithms or improvised version after hyperparameter optimization will be the most suitable for classifying the microbes in general with high accuracy and lowest false alarm rate. The LR was selected for finding if a linear classifier can be used for the said task. The MLP algorithm is based on multi-layered regression equations. The KNN algorithm computes class data points based on distance metrics. The SVM assumes that the correlation and covariance need to be eliminated between the features before it can process. The QDA completes its task of classification without considering covariance as an impediment. By using these models and metrics, it is expected that by the end of the evaluation, a stable and accurate machine model can be constructed using the feedback from the performances of all the algorithms. Principal component analysis (PCA) [67] was used to realize the invariant and orthogonal properties of data [68]. It is expected that this process might lead to the construction of a robust classifier. In the next section, an exhaustive experimentation for selecting appropriate hyperparameters w.r.t. each machine learning model is discussed.

## 3. Results

In this section, the outcome of all the steps taken for automating the process of identifying and classifying microbes are discussed. This section gives detailed information about the iterative progress done for devising modified algorithms that classify ten microbes. Before we discuss further, it must be noted that there are fair chances of bias in the data. Collection of a particular species data may be high due to abundance at sampling stations.

The problem of imbalance in the dataset was not addressed using algorithms that apply sampling from the majority/minority class as it may lead to either overfitting or underfitting of some class data. Loss of information is another reason for adopting methods that rely only on hyperparameter tuning [69] for constructing machine models. It was discovered that scale in terms of geometric properties was different for each microbe. Hence, normalization of the dataset was done using minmax algorithm [70]. This was followed by the comparison of two strategies. The first strategy evaluates the PCA-based dataset for finding the best hyperparameters and the second strategy, uses the mutual information (MI)-based dataset. The mutual information technique was selected after exploration with other feature selection techniques such as p-test, chi-square, t-test, and handcrafted features. These techniques did not impact the accuracy in a positive manner, therefore these methods were dropped for further investigations. Table 6 gives the comparison between PCA- and MI-based models. 

It can be observed from the process of hyperparameter tuning that the use of PCA was not useful. This may be attributed to the fact that there is a loss of information when PCA reduces the dimensions of dataset. Hence, feature selection based on information gain theory was adopted [71]. This was done by estimating mutual information for each discrete target class [72]. As per the definition of mutual information metric [73], it is a measure to calculate dependency between the variables by using non-parametric functions. Non-parametric methods employed entropy estimation with the help of k-nearest neighbors (k = 3) distance. The outcome of this method is shown in Table 7.

It can be observed from Table 7 that the Euler Number, Bounding Box 2, Bounding Box 3, Bounding Box 4, and Convex Hull 1 carried the lowest level of information gain. Therefore, these features were eliminated from the final set of extracted features. The advantage of using this strategy of selecting features for machine learning algorithms is that it considers total correlation and interaction information for giving the degree of dependency between multivariate data. This resulted in the increase of performance of all the algorithms by 1–2% when compared to the PCA-based machine learning model as can be seen from Table 6. Further tests and evaluation on the MI-based machine learning models revealed that the maximum possible accuracy of the best performer algorithm (SVM radial) remains bracketed around 96–97% in terms of accuracy, precision, and recall. The rbf kernel was chosen because it is most frequently used by contemporary researchers [30,74,75]. It would be logical to evaluate the other kernels before moving to the improvisation of the kernel function for improving the performance of the MI-based algorithm. Hence, the polynomial kernel needs to be evaluated as well. It was assessed till eight degrees but it could not improve the accuracy and other performance parameters. Therefore, the next step for building a model with higher accuracy was to design and implement a modified kernel function of SVM. The premise of the improvisation is based on the feedback obtained from the hyperparameter tuning process, especially from the behavior of SVM hyperparameters (gamma, C). In the case of the SVM radial for the MI-based model, the value of gamma = 92 was found by using a random grid-search method. At the same time, it can be observed that the value of C (regularization parameter) or penalty was kept at 4.3. This way, the highest possible level of accuracy (97.2%) was achieved. This is a case of high bias and low variance of data. It allows the classifier to perform with higher numbers of misclassifications. This case implies that the predictions will be similar to one another but on average, they may yield inaccurate results. This condition can be avoided by following two steps. In the first step, modifications in the rbf kernel will be made, hence devising an improvised SVM (ISVM) model. In the second step, multiple validation rounds will be executed to check the consistency of the improvised SVM. The advantage of ISVM is that it removes outliers and extreme values by computing the difference between the first and third quartiles. This way, the values of each feature were brought close to their respective median by subtracting each value with inter quartile range (IQR). Median is a robust measure of the central tendency of any random variable. This step helps to reduce bias in the model as well. The Python script used to implement the ISVM kernel is given in Table 8.

To check the consistency of performance for the modified machine learning model, the experimentation process was further extended by employing 10-fold cross validation. Table 9 gives the values of the performance metrics used for validating the performance of ISVM and SVM. Clearly, it can be observed that the modified rbf kernel was able to achieve 2% higher accuracy as compared to the SVM radial.

The better performance of the ISVM can be attributed to the fact that it takes advantage of the robust statistical method of removing extreme values in each attribute before it transforms the data points for classification. This function was important since our database is imbalance in nature. It is preferred that the classifier must have high true positives or simply high precision as mathematically the precision formula does not consider false negatives. This has come true in our case as shown in Table 9. In addition, if the algorithms give wrong results in terms of classification class, the cost may not be high as in the case of some medical diagnose. Hence, high precision (98.2%) will suffice our purpose. Recall cannot be disregarded even slightly in the context of our ISVM classifier because of the same reason: our algorithm is a generalized classifier for multiple categories of microorganisms. Low recall value will lead to high cost of missing out on a microorganism and at the same time if a fungi is classified as algae or vice versa, it would further affect the entire taxonomical hierarchy.

The accuracy metric considers the values of true positives and true negatives while the F1-score [76] helps to make crucial facts that arise due to the values of false negatives and false positives. Since, the F1-score is the harmonic mean of recall and precision, it gives a better idea about the incorrectly classified cases. The F1 score also becomes important when the distribution of class instances is not equal, as is evident in our case. It can be observed from Table 9 that 10-fold cross validation done on the basis of the F1-score shows a high value of 98.1%. From all these values, it can be safely concluded that ISVM is the most appropriate algorithm for building a generalized classifier for water surface microbes.

## 4. Discussion

It can be inferred from this research that it is a difficult task to create a generalized segmentation algorithm for microorganisms that work well for almost all forms of microorganisms, such as fungi, algae, and Eukaryotes. Filters such as Kirsch have been found to function best in defining and extracting the boundaries of microorganisms. Its combination with global pixel thresholding techniques such as Otsu improved the accuracy of the segmentation algorithm. In the context of our research, the primary task of the Otsu algorithm was to cluster pixels that are on the edges of each microorganism and isolate it from its inner body parts. The generalized segmentation algorithm (GSA) has been validated by IoU metrics.

An algorithm can only be used for real time if its performance has been validated. Therefore, in this research, we have used multiple methods to validate each process that makes the automation of microorganisms stable. For selecting the most accurate and stable classifier, the model selection process with the help of a 10-fold algorithm was done. This ensured that there is no wastage of time in evaluating multiple machine learning models at a later stage. It was found that the usage of PCA for feature engineering and dimension reduction does not provide any additional advantage and it leads to loss of information. Hence, a mutual information-based feature selection model was adopted which allowed us to construct an optimized SVM radial model. However, the hyperparameter tuning report showed that the SVM radial kernel required some improvisation because the SVM radial did not improve its accuracy beyond 97% with the best possible hyperparameters. The modified rbf kernel with the IQR method provided a higher level of performance. It can safely be concluded that the strategy followed here yielded excellent results because, in our research work, domain experts were also available. With the help of domain experts, the tedious process of manual identification and grouping of microorganisms was simplified. In the absence of a domain specialist, this work would have required additional resources and a new stack of algorithms such as active learning [77] and deep learning [78].

## 5. Conclusions

In summary, it can be stated that this work is an amalgamation of a newly constructed microbe segmentation algorithm and an improvised version of the support vector machine. The generalized segmentation algorithm (GSA) facilitated the generation of high-quality inputs for machine learning models. This is stated based on rigorous evaluation for the segmentation process which empirically showed that GSA acquired an overall accuracy of 90% in step 1 (Kirsch filter) and 88% in step 2 (Otsu clustering). An exhaustive grid search method of hyperparameter tuning ensured that a highly optimized automation process is adopted. Mutual information feature selection proved to be a very useful technique for selecting the best possible features and it overcame the inadequacies of PCA and other feature selection methods. From the performance of all the machine learning algorithms, it became imperative to modify the SVM algorithm as it could only acquire maximum accuracy of 96.1%. Hence, improvisation of the rbf kernel was done with the help of the inter quartile range (IQR). The whole strategy yielded a highly accurate classifier model with an accuracy of 98.2%. The result of this research work i.e., improvised support vector machine model (ISVM) can be further serialized with libraries such as joblib. With the help of a serialized dump of the model, the algorithm can be deployed in the cloud ML (machine learning) engines such as Google, Amazon Web Services, etc. In the future, it is suggested that in case the number of instances for microbe images are less, transfer learning [79] may be adopted.

## Figures and Tables

**Table 1:** List of microorganisms automated using classifiers.

S.No	Microorganism Class	Microorganism Type	Number of Instances
1	Spirogyra	Algae	4012
2	Volvox	Algae	7002
3	Phithophora	Algae	2303
4	Yeast	Fungi	4302
5	Rhizopus	Fungi	3910
6	Penicillium	Fungi	3410
7	Aspergillus sp	Fungi	3230
8	Protozoa	Eukaryotes	1230
9	Diatom	Algae	1450
10	Ulothrix	Algae	1930
		Total	32,779

**Table 2:** Selection of convolution filters based on IoU.

S.No	Convolution Filter	Accepted/Sample			Average Accuracy
		25	50	75	
1	Prewitt Filter [47]	20	23	39	0.59
2	LOG Filter [32]	13	31	39	0.55
3	Laplacian Filter [48]	15	39	67	0.76
4	Low Pass Gaussian [47]	14	38	66	0.73
5	Sobel Filter [32]	19	36	69	0.80
6	Mean Filter [49]	20	40	68	0.84
7	Kirsch Filter [50]	22	45	70	0.90

**Table 3:** Comparison of clustering algorithms.

	Evaluation Round	Sample Size ‘S’	Accuracy (AC/S)	Average Accuracy
Otsu	1	10	1	0.88
	2	30	0.93	
	3	40	0.90	
	4	50	0.79	
	5	100	0.79	
ISO Data	6	10	1	0.81
	7	30	0.83	
	8	40	0.90	
	9	50	0.62	
	10	100	0.73	

**Table 4:** Output of the GSA algorithm.

Microbe	Original	Contrast Enhanced	Microbe Body Extracted	Segmented Image
Spirogyra	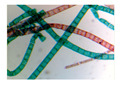	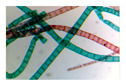	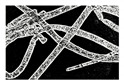	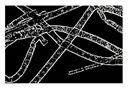
Pithophora	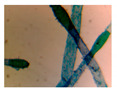	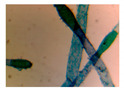	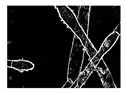	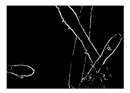
Yeast	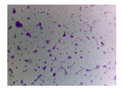	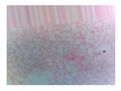	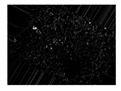	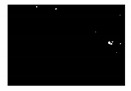
Raizopus	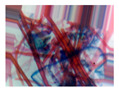	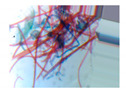	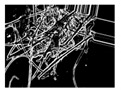	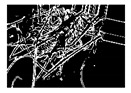
Penicillum	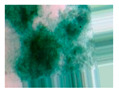	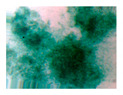	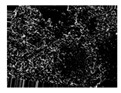	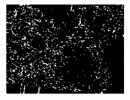
Aspergillus sp	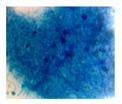	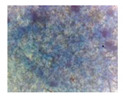	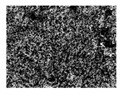	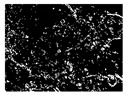
Protozoa	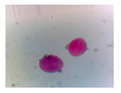	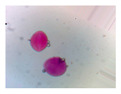	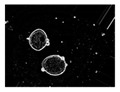	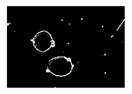
Diatom	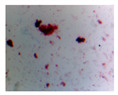	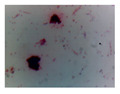	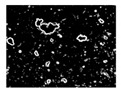	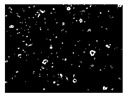
Ulothrix	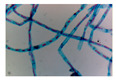	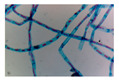	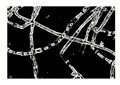	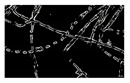

**Table 5:** Formulae of evaluation parameters.

Metric	Formula
Precision [60]	$\frac{True\;Positive}{True\;Positive+False\;Positive}$
Recall [60]	$\frac{True\;Positive}{\left(True\;Positive+False\;Negative\right)}$
Accuracy [60]	$\frac{True\;Positive+True\;Negative}{Positive+Negative}$
F1-Score [61]	$\frac{2*Precision*TruePositiveRate}{Precision+TruePositiveRate}$

**Table 6:** Hyperparameter optimization (PCA-based model vs. MI-based model): A = Accuracy (%), P = Precision (%), R = Recall (%).

Model	Hyper Parameters and Their Ranges	Best Hyper Parameters Found (PCA)	Best Hyper Parameters Found (MI)	PCA			MI		
				A	P	R	A	P	R
LR	Penalty: ‘l2’; Solver: [‘newton-cg’,’lbfgs’,’liblinear’, ‘sag’, ‘saga’]	penalty: l2 Solver: newton-cg	penalty: l2 Solver: newton-cg	23.1	5.1	23.2	24.9	5.9	24.9
KNN	n_neighbors: 1–15; Weights: [‘uniform’, ‘distance’]; Leaf Size: [1, 3, 5]; Algorithm: [‘auto’, ‘kd_tree’]	n_neighbors: 3,Weights: distance, leaf_size: 3, algorithm: auto	n_neighbors: 3, Weights: distance, leaf_size: 3, algorithm: kd tree	96.1	96.1	96.2	96.1	96.1	96.0
SVM radial	gamma: log(−2, 2, 5); C: log(−2, 2, 5)	gamma: 100.0, C: 1.0	gamma: 92.0, C: 4.3	96.2	96.3	96.3	97.2	97.3	97.0
MLP	hidden layer sizes: [(10–50)]; Activation: [‘identity’, ‘logistic’, ‘tanh’, ‘relu’]; Solver: [‘lbfgs’, ‘sgd’, ‘adam’]; Alpha: log(−5, 3, 5); Learning Rate: [‘constant’, ‘invscaling’,’adaptive’]; Max Iteration: [100, 500, 1000]	hidden_layer_sizes: 50, activation: relu, solver: lbfgs, alpha: 0.001, learning_rate: constant, max_iter: 1000	hidden_layer_sizes: 50, activation: relu, solver: adam, alpha: 0.002, learning_rate: constant, max_iter: 1000	29.6	26.8	29.7	31.2	27.8	31.7
QDA	priors: [None]; reg_param: (0 0.1 0.2 0.3 0.4 0.5 0.6 0.7 0.8 0.9)	priors: None, reg_param: 0.2	priors: None, reg_param: 0.3	24.8	10.1	24.8	26.8	11.1	26.4

**Table 7:** Estimated mutual information for feature selection.

Feature Name	Estimated Mutual Information
Solidity	1.686472
Eccentricity	1.669454
EquivDiameter	1.576661
Extrema	1.091982
Filled Area	1.606655
Extent	1.697518
Orientation	1.723908
**Euler Number**	**0.539431**
Bounding Box 1	1.091185
**Bounding Box 2**	**0.970946**
**Bounding Box 3**	**0.656073**
**Bounding Box 4**	**0.666515**
**Convex Hull 1**	**1.078976**
Convex Hull 2	1.08001
Convex Hull 3	1.109029
Convex Hull 4	1.18575
Major Axis	1.748819
Minor Axis	1.744697
Perimeter	1.735635
Convex Area	1.68434
Centroid 1	1.744533
Centroid 2	1.721187
Area	1.578785
Radii	2.10396

**Table 8:** Python script of the modified rbf kernel function.

Python Script	Description
def apply_IQR(X): [q1,q2,q3] = ComputeQuartiles(X) iqr = (q3 − q1) return X-iqr	The value of IQR is computed on the basis of quartiles. It is basically the difference between the q3 and q1. Each quartile is a median computed using the following rules: Given an even 2n or odd 2n + 1 number of values, first quartile Q1 = median of the n smallest values, third quartile Q3 = median of the n largest values. The second quartile Q2 is the same as the ordinary median.
def modified_rbf(X,Y,IQR = True): Xm = Apply_IQR(X) K = np.zeros((Xm.shape[0],Y.shape[0])) for i,x in enumerate(Xm): for j,y in enumerate(Y): K[i,j] = np.exp(-1*np.linalg.norm(x-y)**2) return K	This is the definition of the modified rbf kernel in which; first IQR is computed for each feature and then the rbf equation is applied.

**Table 9:** Performance of SVM vs. ISVM.

Model	Accuracy (%)	Precision (%)	Recall (%)	F1-Score (%)
SVM	96.1	96.2	96.1	96.1
ISVM	98.2	98.2	98.1	98.1

## Data Availability

The data presented in this study are openly available in [Mendeley Data] at [doi: 10.17632/f9m85ptmvc.4].
